# *HOTAIR*: an oncogenic long non-coding RNA in different cancers

**DOI:** 10.7497/j.issn.2095-3941.2015.0006

**Published:** 2015-03

**Authors:** Mohammadreza Hajjari, Adrian Salavaty

**Affiliations:** Department of Genetics, Faculty of Science, Shahid Chamran University of Ahvaz, Ahvaz 61336-3337, Iran

**Keywords:** *HOTAIR*, long non-coding RNA (lncRNA), epigenetic, cancer

## Abstract

Long non-coding RNAs (lncRNAs) refer to a group of RNAs that are usually more than 200 nucleotides and are not involved in protein generation. Instead, lncRNAs are involved in different regulatory processes, such as regulation of gene expression. Different lncRNAs exist throughout the genome. LncRNAs are also known for their roles in different human diseases such as cancer. *HOTAIR* is an lncRNA that plays a role as an oncogenic molecule in different cancer cells, such as breast, gastric, colorectal, and cervical cancer cells. Therefore, *HOTAIR* expression level is a potential biomarker for diagnostic and therapeutic purposes in several cancers. This RNA takes part in epigenetic regulation of genes and plays an important role in different cellular pathways by interacting with Polycomb Repressive Complex 2 (PRC2). In this review, we describe the molecular function and regulation of *HOTAIR* and its role in different types of cancers.

## Introduction

The physiological and developmental complications of humans lead us to the point that the limited number of protein-coding genes compared with whole genome cannot explain the complexity of human features^1^. Most of the genomic DNAs (at least 70%-90%) are transcribed to the RNAs that do not produce any proteins. These parts of the genomes are known as non-coding RNA (ncRNA) genes, which produce efficient RNA molecules^1–4^.

LncRNAs consist of a group of ncRNAs, including thousands of various species^5^. These RNAs are usually more than 200 nucleotides and are mostly transcribed by RNA pol II from different regions across the genome. They play different roles such as transcriptional and post-transcriptional regulation inside the cell. Recently, lncRNAs are known as key regulators of gene expression^6–8^. By working on embryonic stem cells and through analyzing the ribosome profiling data, Chew *et al*.^9^ revealed that many lncRNAs are protein-coding contaminants. LncRNAs also regulate the activity of epigenetic machinery during cell differentiation^10^. In fact, many lncRNAs recruit chromatin-modifying proteins (e.g., PRC2) to specific sites of genome and affect gene expression through regulating chromatin states^11^.

Based on their roles, the dysregulation of lncRNAs is involved in several diseases including cancer^12,13^. Du *et al*.^14^ analyzed the expression of different lncRNAs in various tumors. Through this analysis, they identified the lncRNAs related to different cancers and their clinical prognosis. Dysregulation of lncRNAs is related to prognosis, metastasis, and recurrence in different cancer types. Studies show that dysregulation of certain lncRNAs affect several processes related to oncogenesis, including cell growth and proliferation^15^. The over expression of some lncRNAs with proto-oncogenic function in normal cells increases tumor growth and matrix invasion of cancer cells^14,16^. Moreover, over-expression of oncogenic lncRNAs results in tumor-cell proliferation and metastasis through chromatin looping and some other processes^17^.

In this review, we describe the oncogenic roles of *HOTAIR* long-non coding RNA as one of the most important regulatory RNAs in human cells. We also present the molecular function and regulation of this lncRNA in different types of cancer.

## *HOTAIR* lncRNA

*HOTAIR* lncRNA was introduced by Rinn *et al*.^18^ as a spliced and polyadenylated RNA with 2,158 nucleotides and 6 exons. This RNA arises from the transcription of antisense strand of *HoxC* gene, which is specifically situated between *HoxC11* and *HoxC12* on chromosome 12q13.13. Computational and Northern blot analysis revealed that *HOTAIR* does not show any stem loops suggestive of being a pre-miRNA. These analysis also suggested that *HOTAIR* is preferentially expressed in posterior and distal sites of the human body. In an experiment on 10 mammalian genomes and 3 non-mammalian vertebrates, He *et al*.^19^ looked for matches to the 6 exons of *HOTAIR* and its two conserved domains. They reported a poor sequence conservation and, by contrast, noticeably conserved structures for *HOTAIR*. They also reported that *HOTAIR* has evolved faster compared with adjacent HoxC genes.

**Figure 1:**
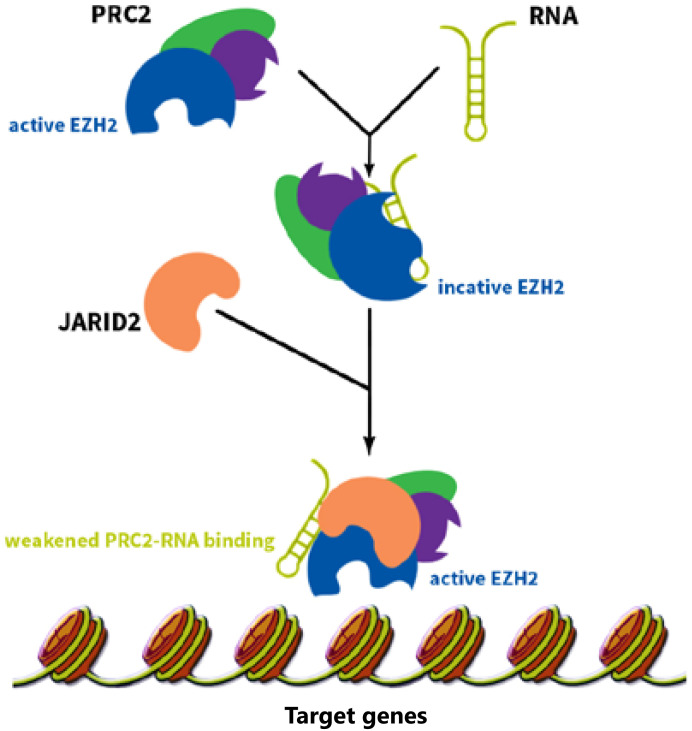
The RNAs recruiting PRC2 complex inhibit PRC2 function. These RNAs guide PRC2 to its target gene and inhibits EZH2 enzymatic activity at the same time. When PRC2 reaches its target gene, another protein called JARID2 comes into play and binds to EZH2, weakens EZH2-RNA binding, and consequently activates EZH2’s function.

**Figure 2:**
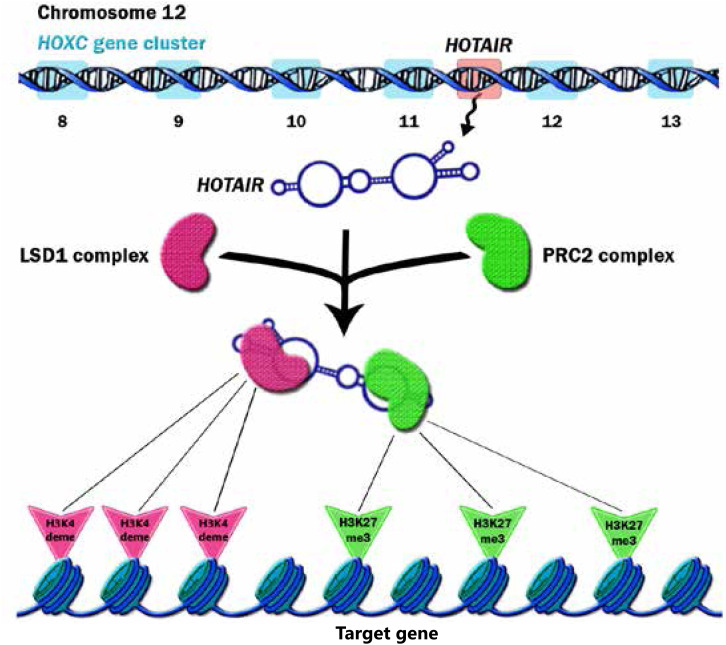
*HOTAIR* gene is located on chromosome 12 inside the *HoxC* locus, specifically between *HoxC11* and *HoxC12*. After the expression of *HOTAIR*, this lncRNA recruits PRC2 and LSD1 complexes and thus functions as a bridge. *HOTAIR* directs these complexes to their target genes and as a result regulates the trimethylation of H3K27 and demethylation of H3K4 at targeted genes. H3K27me3 and H3K4deme refer to the trimethylation of histone H3 at lysine-27 and the demethylation of histone H3 at lysine 4, respectively.

*HOTAIR* is a trans-acting lncRNA and has different target loci such as HOXD^4^. *HOTAIR* interacts with Polycomb Repressive Complex 2 (PRC2) and is necessary for PRC2 occupancy and histone H3 lysine-27 trimethylation of different genes in different chromosomes. PRC2 is a histone methyltransferase that implements epigenetic silencing during different processes including cancer development^20^. *HOTAIR* localizes and targets PRC2 genome wide^21^. PRC2 is a complex that contains three major subunits, including EZH2, SUZ12, and EED. Although EZH2 is the key player for the methyltransfer process, other subunits are also required to regulate EZH2 catalytic activity^22^. The affinity of EZH2 to RNA is regulated by EED, which increases the specificity of PRC2 function. Cifuentes-Rojas *et al*.^23^ investigated the PRC2-RNA interaction precisely. They showed that RNA directs PRC2 to its target gene and simultaneously inhibits the enzymatic activity of EZH2. When PRC2 reaches its target gene, JARID2 binds to EZH2 to impair PRC2’s binding to RNA and thereby activates EZH2’s function (**Figure 1**). Knockdown of JARID2 results in reduction of H3K27me3 levels on some target genes^24^. JARID2 also may have a negative impact on PRC2’s function, and deletion of JARID2 results in the enhancement of H3K27me3 levels on some target genes^25^. *HOTAIR* functions as a molecular scaffold and interacts not only with PRC2 but also with LSD1 complex to regulate gene expression. LSD1 involves in demethylation of histone H3 at lysine 4^26,27^ (**Figure 2**). Specifically, PRC2 binds to a 5' domain and LSD1 to a 3' domain of *HOTAIR*, and *HOTAIR* coordinates their functions for chromatin modification^28^. Through these functions, *HOTAIR* affects the expression of multiple genes involved in various cellular functions^21^.

## *HOTAIR* lncRNA as an oncogenic factor in different cancers

*HOTAIR* is an oncogenic factor and can be used as a prognostic biomarker in different cancer types^29^. *HOTAIR* lncRNA plays a key role in the initiation and progression of different types of cancer such as cervical cancer and nasopharyngeal carcinoma^30,31^. *HOTAIR* also plays an important role in promoting malignancy^32^. To assess the association between *HOTAIR* expression levels and lymph node metastasis, Cai *et al*.^33^ in a meta-analysis surveyed a total of 748 patients from 8 studies. In this meta-analysis, they showed that the patients with high *HOTAIR* expression level had a higher incidence compared with that in patients with low *HOTAIR* expression level. Moreover, Alves *et al*.^34^ investigated the role of *HOTAIR* in epithelial-to-mesenchymal transition (EMT) and also its role in arising and maintenance of cancer stem cells (CSCs). They revealed that *HOTAIR* plays an important role in the process of tumorigenicity by triggering EMT and acquiring stemness.

In fact, *HOTAIR* is involved in several processes associated with carcinogenesis such as those affecting the mobility, proliferation, apoptosis, invasion, aggression, and metastasis of the cells (**Table 1**). PRC2 and LSD1 complexes to exert epigenetic modifications and suppressing a number of genes such as tumor and metastasis suppressor genes. Given these crucial functions, *HOTAIR* is applied as a potential biomarker of various human cancers. In addition, measuring the expression level of *HOTAIR* can help us detect the progression stage of cancer and predict the survival possibility of an individual^21,27,38,39^. Furthermore, *HOTAIR* is involved in the resistance of cancer cells to cisplatin. This role of *HOTAIR* is at least attributed to the downregulation of *P21* gene. Liu *et al*.^60^ found that knockdown of *HOTAIR* could resensitize the responses of A549/DDP cells to cisplatin. Interestingly, different functional SNPs across whole *HOTAIR* locus have been reported to influence the cancer risk^61^.

**Table 1:** Overexpression of *HOTAIR* in different cancers

Type	Overexpression of *HOTAIR*	References
Breast cancer	Poor prognosis, metastasis, invasion, and short overall survival	^21^,^35^
Esophageal squamous cell carcinoma (ESCC)	Poor prognosis, high TNM stage, invasion, metastasis, and short overall survival	^36^,^37^
Gastric cancer	Tumor staging, venous infiltration, and lymph node metastasis	^38^,^39^
Hepatocellular carcinoma	Invasion of HCC cells, possibility of recurrence	^40^–^44^
Colorectal cancer	Poor prognosis, low survival, and metastasis promotion	^45^–^47^
Gallbladder cancer (GBC)	Promoting carcinogenesis	^29^
Bladder cancer (BC)	Poor prognosis and high recurrence rate	^48^
Renal carcinoma	Proliferation, invasion, and promotion of tumor growth	^49^
Cervical cancer	FIGO stage, aggression, and lymph node metastasis	^30^
Epithelial ovarian cancer	Poor prognosis, FIGO stage, lymph node metastasis, overall survival, and metastatic stage of EOC	^50^
Endometrial carcinoma	Poor prognosis, lymph node metastasis, EC grade, and overall survival	^51^,^52^
Lung cancer	Invasion and metastasis	^53^
Non-small cell lung cancer	Promotion of lymph node metastasis	^54^,^55^
Small-cell lung cancer	Poor prognosis, proliferation and invasion	^56^
Nasopharyngeal carcinoma	Poor prognosis, overall survival, proliferation, invasion, and promotion of tumor stage	^31^
Melanoma	Invasion and metastasis	^57^
Glioma	Poor prognosis, cell cycle progression, and glioma grade	^58^
Pancreatic cancer	Proliferation and aggression of tumors	^59^

## Regulation of *HOTAIR* through different pathways

The expression level of *HOTAIR* gene and the function of its transcript can be controlled by several factors (**Table 2**). The DNA methylation pattern of downstream intergenic CpG island of *HOTAIR* may have an important effect on its expression level^62^. Moreover, the post-synthetic methylation of some cytosines of *HOTAIR* has been reported. This post-synthetic methylation within or near important functional regions of *HOTAIR* may play an important role in the regulation of *HOTAIR* function^4^.

**Table 2:** *HOTAIR* regulatory factors

Factors	Up/down-regulation	Regulatory level	References
Methylation of downstream intergenic CpG island	Downregulation	Transcriptional	^62^
Post-synthetic methylation	Downregulation	Post-transcriptional	^4^
Functional SNPs across *HOTAIR* locus	Up/downregulation	Transcriptional/ post-transcriptional	^61^
siRNA	Downregulation	Post-transcriptional	^49^,^51^
MiR-141	Downregulation	Post-transcriptional	^32^
Argonaute2 (Ago2)	Downregulation	Post-transcriptional	^32^
Osteopontin (OPN)	Upregulation	Transcriptional	^63^
IRF1	Downregulation	Transcriptional	^63^
c-Myc	Upregulation	Transcriptional	^29^
TGF-β	Upregulation	Transcriptional	^4^
Diethylstilbestrol (DES)	Upregulation	Transcriptional	^64^
Bisphenol-A (BPA)	Upregulation	Transcriptional	^64^
Estrogen receptors (ERs) and ER coregulators	Upregulation	Transcriptional	^64^
Type I collagen (Col-1)	Upregulation	Transcriptional	^65^

The function of *HOTAIR* can be suppressed by argonaute2 (Ago2) complex in the presence of microRNA-141 (*miR-141*). *MiR-141*, unlike *HOTAIR*, is a suppressor of tumorigenicity, invasiveness, and malignancy in several cancer types. *MiR-141* first binds to *HOTAIR* to suppress it, and Ago2 complex comes into play and cleaves the *HOTAIR*^32^.

A type of phosphoglycoprotein called osteopontin (OPN), which is an extracellular matrix protein, can transcriptionally activate and increase *HOTAIR* expression in cancer cells. Receptor CD44, a positive regulator of OPN, affects the expression level of *HOTAIR*. By contrast, interferon regulatory factor 1 (IRF1) decreases *HOTAIR* expression level by binding to its promoter. In fact, OPN regulates IRF1 and affects its signaling pathway, thus activating *HOTAIR* expression by suppressing the function of IRF1^63^.

The protein c-Myc is another element that impacts the expression of *HOTAIR*. c-Myc is an oncoprotein that plays a role in the development of several types of cancer through regulating several protein-coding and non-coding genes. c-Myc recognizes a putative E-box element in the upstream region of *HOTAIR*, which is approximately located at the 1,053 upstream within its promoter. c-Myc directly interacts with this E-box element and upregulates the expression of *HOTAIR*. In addition, knockdown of c-Myc can reduce both *HOTAIR* expression and its promoter activity, whereas upregulation of c-*Myc* gene increases *HOTAIR* expression and its promoter activity^29^. Moreover, silico analysis identified four potential Myc-binding sites within *HOTAIR* promoter^4^.

Researchers working on human breast cancer cells have shown that diethylstilbestrol and bisphenol-A can upregulate the expression of *HOTAIR* in these cells^66^. Some evidence showed the existence of estrogen response elements in the promoter of *HOTAIR*. Estrogen receptors (ERs) and ER coregulators, such as histone methylases mixed lineage leukemia (MLL) 1, MLL3, and CREB-binding protein/p300, induce the expression of *HOTAIR* by binding to its promoter^64^.

TGF-β is another factor that induces *HOTAIR* expression and involves in EMT, which results in arising and maintenance of CSCs^4^. Furthermore, evidence suggests the effect of type I collagen (Col-1) on *HOTAIR* upregulation. Zhuang *et al*.^65^ showed that Col-1, which is aberrantly enriched in the tumor microenvironment, can induce the expression of *HOTAIR* in lung cancer cells^53^. In addition, *HOTAIR* overexpression has been reported in non-small cell lung cancer^54^.

## *HOTAIR* functions

### Coordination with PRC2

Li *et al*.^26^ showed that directed deletion of *HOTAIR* lncRNA in mouse can result in activation of hundreds of genes. Different downstream pathways and genes are attributed to the molecular roles of *HOTAIR* in human cells. *HOTAIR* can act through promoting the chromatin relocalization done by PRC2. This targeting of PRC2 in the genome leads into a distinct pattern of gene expression necessary for breast cancer progression^21,35^. Specifically, an overlap exists between *HOTAIR*-binding motif and BRCA1-binding region in EZH2. This finding indicates that decreased expression of BRCA1 results in elevated recruitment of PRC2 by *HOTAIR* in breast cancer cell lines^4^.

### *HOTAIR* functions through Wnt/β-catenin

The key role of *HOTAIR* in the development and progression of esophageal squamous cell carcinoma (ESCC) has been revealed^36^. *HOTAIR* exerts its role through activating Wnt/β-catenin signaling pathway. The Wnt/β-catenin signaling pathway is an important pathway in the development of ESCC. *HOTAIR* recruits PRC2 directly to the promoter region of *Wnt inhibitory factor 1* (*WIF-1*), leading to the reduction of *WIF-1* expression and consequently the activation of Wnt/β-catenin signaling pathway^37,61^. In addition, different functional SNPs across the whole *HOTAIR* locus that affects the regulation of *HOTAIR* may influence ESCC risk^61^.

### Involvement in EMT

Through a series of *in vitro* and *in vivo* assays on epithelial ovarian cancer (EOC) tissues, researchers showed that a significant association exists between *HOTAIR* expression level and metastatic stage of EOC. This association may be due to the regulation of certain matrix metalloproteinases (MMPs) and EMT-related genes by *HOTAIR*. *HOTAIR* expression is also associated with FIGO stage/metastasis of lymph nodes; thus, this factor could be a potential biomarker or therapeutic target in EOC patients^50^.

Inhibition of *HOTAIR* in gastric cancer cells leads to EMT process reversal and reduction of invasiveness mediated by the expression of MMP1 and MMP3^67^. Some evidence shows that suppression of *miR-7* by *HOTAIR* can mediate EMT progression in breast cancer. This microRNA can inhibit the SETDB1 and STAT3 pathway in breast cells^68^.

### Functions as competitive endogenous RNAs

*HOTAIR* can function as competitive endogenous RNAs (ceRNAs) in gastric cells by recruiting the microRNAs targeting the HER-2. Thus, *HOTAIR* and HER-2 may have coexpression in gastric cancer tissues^69^. Given this recently identified role of *HOTAIR*, the finding indicative of the positive interaction between *HOTAIR* and HER2 is worth to research in other types of cancer cells.

*miRNA-130a* binding sites were found in *HOTAIR* lncRNA. A negative correlation between *HOTAIR* and *miRNA-130a* has been demonstrated in gallbladder cancer tissues compared with nearby normal tissues. Thus, the oncogenic role of *HOTAIR* is not only by recruiting PRC2 but also partly through negative regulation of *miRNA-130a*^29^.

### *HOTAIR* regulates various genes in tumors

*HOTAIR* overexpression is also related to hepatocellular carcinoma (HCC)^40–43^. In an experiment, Ding *et al*.^44^ showed that suppression of *HOTAIR* leads to the increase of RNA binding motif 38 (RBM38) proteins, which play a role in the regulation of cell motility. They also showed that RBM38 expression levels were lower in HCC tissues compared with noncancerous tissues in the same patients. Therefore, *HOTAIR* increases the aggression and invasion of HCC cells by suppressing RBM38 expression.

Some reports indicate that *HOTAIR* plays an oncogenic role partially via the downregulation of *HOXA5*^55^. The results also suggest that this lncRNA plays a potential oncogenic role through influencing the expression of specific genes associated with cell adhesion such as *MUC5AC* and *ASTN1*^56^. Kogo *et al*.^45^ suggested that *HOTAIR* regulates the expression of multiple genes in cooperation with PRC2 and raises the levels of undifferentiated cancer cells in CRC patients.

In an experiment, Yan *et al*.^48^ measured the *HOTAIR* expression level of Ta/T1 bladder cancer tissues and adjacent normal tissues, which were collected from 110 patients. They reported that 90 specimens had high *HOTAIR* expression levels, which were inversely correlated with *WIF-1* expression. *HOTAIR* may also be involved in the development of colorectal cancer^46,47^.

Wu *et al*.^49^ reported an increase in *HOTAIR* expression level in renal carcinoma cells. They also reported that knockdown of *HOTAIR* by siRNA impacts the cell cycle in the G_0_/G_1_ phase and also decreases the cell proliferation and invasion *in vitro*. These effects resulted in the reduction of *HOTAIR* binding ability to EZH2 and consequently the reduction of H3K27me3 on *HOTAIR* target genes. Furthermore, they showed that inhibition of *HOTAIR* expression resulted in suppression of growth of xenograft tumors formed by renal carcinoma cells. In addition, they demonstrated that inhibition of *HOTAIR* expression and modulation of covalent histones activated transcriptional state of cell cycle-related gene.

Tang *et al*.^57^ investigated the potential roles of *HOTAIR* in melanoma cells and showed that *HOTAIR* is overexpressed in metastatic melanoma tissues. They showed that knockdown of *HOTAIR* by siRNAs resulted in the reduction of motility and invasion of human melanoma cell line A375. They also reported that *HOTAIR* is involved in promoting gelatinase activity in melanoma cells.

Different studies revealed that increasing the expression of *HOTAIR* has several effects, such as increasing the proliferation and aggression of cancer cells in pancreatic cancer tissues. On the contrary, downregulation of *HOTAIR* has opposite effects, including inhibition of cell cycle progression, reducing proliferation, and increasing apoptosis. However, the results of gene array studies showed that some differences exist between *HOTAIR*-regulated genes in pancreatic cells and breast cancer cells. These studies suggest several interferon- and cell cycle-related genes as targets of *HOTAIR* in pancreatic cancer cells^59^. Furthermore, overexpression of *HOTAIR* may be associated with endometrial carcinoma^51,52^.

Although the exact roles of *HOTAIR* in glioma and glioblastoma are still challenging, *HOTAIR* can be used as a prognostic biomarker in glioma^58^.

## Conclusion

Different studies provide some evidence for the crucial roles of *HOTAIR* in the initiation and progression of various cancers. Understanding the biological roles of *HOTAIR* in different types of cancer helps us to determine the efficiency of this lncRNA as a diagnostic or predictive biomarker. However, *HOTAIR* has been suggested as a biomarker for most cancer types. Thus, conducting a meta-analysis of *HOTAIR* expression in all cancer types may help in the identification of the cancers that have the highest probability of *HOTAIR* overexpression. Hence, *HOTAIR* expression level can be used as a potential prognostic factor for various cancers. In addition, screening the *HOTAIR* overexpression can help us identify cancer progression and tumor stage. Expression analysis can be conducted by different quantitative techniques such as RT-PCR. These assays would be valuable when the RNA level can be differentially analyzed in samples of patients such as urine, blood, and mucus. However, clinical trials are needed in the future to find this RNA as a suitable biomarker or therapeutic target in cancer.

*HOTAIR* can also be further considered as a therapeutic target to improve the sensitivity of therapy for different tumors. Moreover, the efficacy of these therapeutic approaches can be further expanded via identification of exact molecular pathways underlying the regulation of *HOTAIR* expression. Although some regulatory pathways of *HOTAIR* expression have been reported, thorough identification of those pathways requires more studies and experiments.
